# Editorial: Stress-mediated regulation of plant specialized metabolism

**DOI:** 10.3389/fpls.2023.1290281

**Published:** 2023-09-22

**Authors:** Sonal Mishra, Shakti Mehrotra, Vikas Srivastava

**Affiliations:** ^1^ Department of Botany, School of Life Sciences, Central University of Jammu, Samba, Jammu and Kashmir, India; ^2^ Department of Biotechnology, Institute of Engineering and Technology, Dr. A.P.J. Abdul Kalam Technical University, Lucknow, India

**Keywords:** secondary metabolites, transcription factors, environmental stress, gene, metabolism

Plant specialised metabolites (PSM or secondary metabolites) are species-specific metabolites, responsible for successful endurance of plants under diverse ecological conditions, and helpful for resistance to biotic and abiotic stress (Marone et al., 2022; Huang and Dudareva, 2023). Based on their biosynthesis, PSMs have been classified into many classes viz. alkaloids and nitrogen-containing compounds, terpenoids, phenolics, benzenoids, and phenylpropanoids, indole and sulfur-containing indole compounds, glucosinolates, and fatty acid derivatives (Garagounis et al., 2021). The PSMs are known for their diverse application in human welfare viz. medicine, flavour and fragrance, nutraceutics, cosmetics etc (Kallscheuer et al., 2019; Krasteva et al., 2021; Bagal et al., 2023). These metabolites are usually in limited abundance in native plants; therefore, many biotechnology-based strategies were explored to enhance their production (Srivastava et al., 2018; Chandran et al., 2020; Srivastava et al., 2020; Wu et al., 2021). Nevertheless, it has been also observed that PSMs production is largely regulated by plants’ internal and external cues, which provide an effective strategy to stimulate their production. Stimulation of PSMs have been reported in diverse stress conditions (Mishra et al., 2013; Mishra et al., 2015; Radwan et al., 2017; Yahyazadeh et al., 2018; Thakur et al., 2019), suggesting a positive correlation between environment and metabolite production. The stress response in plants trigger a cascade of signals that work one after other and finally lead to the stimulation of genes associated with PSM biosynthesis, which often provide resistance to the plants (Goyal et al., 2012). However, the method of stress-influenced metabolite changes looks promising to meet the commercial expectation, not require much optimization and easily applicable in field. In the Research Topic, we have attempted to explore the regulation of PSMs under stress situations.

The Research Topic includes seven articles comprised of five original research and two review articles. Four of them, includes study related to transcription factors. The transcription factors have been explored widely in the regulation of plant development, metabolism, and environmental stress (Srivastava et al., 2022). Wu et al. highlighted the significance of Ethylene Responsive Factor (ERF) subfamily in abiotic stress and regulation of genes associated with stress response and flavonoid biosynthesis. Furthermore, Kajla et al. have reviewed the significance of transcription factors (WRKY, bHLH, MYB, NAC, AP2/ERF, and bZIP) in the regulation of plant secondary metabolism during biotic stress and their regulation by miRNAs. In another study, sixty-five *bZIP* transcription factor were explored in the genome of *Isatis indigotica*. The correlation study of transcriptomic and metabolomic data for *I. indigotica* leaves exposed to salt stress, suggested association of *IibZIP23, IibZIP38* and *IibZIP51* with three alkaloids (quinoline alkaloid stylopine, indole alkaloids tabersonine and indole-3-acetic acid), flavonoid myricetin 3-O-galactoside, and two primary metabolites 2-hydroxy-6-aminopurine, 3-dehydroshikimic acid (Jiang et al.). Another study investigated the TEOSINTE BRANCHED1/CYCLOIDEA/PCF (TCP) transcription factor in *Catharanthus roseus* and reported 15 CrTCPs, which were classified further. The UV and methyl jasmonate treatments significantly increased the production of terpene indole alkaloids (TIAs-vindoline, catharanthine and ajmalicine). Moreover, few *CrTCPs* exhibited significant modulation under these conditions, and possess TCP-binding elements in the promoters of TIA-biosynthesis related genes (Hao et al.). A study in Qingke (Tibetan hulless barley) demonstrated genome-wide association studies (GWAS) in several varieties having different tolerance to drought conditions. The metabolic profiling suggested substantial changes in drought-sensitive varieties as compared to drought-resistant qingkes (Yu et al.). The effect of few salts at different concentration in *Mentha longifolia* demonstrated several morphological and physio-biochemical effects. The GC-MS analysis reported higher essential oils at lower-level salt condition, predominated by (−)-carvone and D-limonene, which was also supported by expression studies (Singh et al.). The problem of steroidal glycoalkaloids (SGA) during post-harvest stress (wounding and light) in potato was studied by Merino et al. The study through metabolomic and transcriptomic analysis has identified key genes, the expression of which correlate with SGA differences between cultivars. The overexpression of two (*StHMGR1_MB_* and *StTAM2_MB_*) of them, increased the leaf SGA levels in low-SGA producing cultivar.

In a nutshell, the Research Topic has attempted to include the stress-mediated regulation of plant specialised metabolism and underlying mechanism. The knowledge is important for improving the production of high value therapeutic metabolites, by opting low-cost methods.

## Author contributions

SMi: Writing – original draft, Writing – review & editing. SMe: Writing – review & editing. VS: Conceptualization, Writing – original draft, Writing – review & editing.
